# Antimicrobial Activity of Metal-Based Danofloxacin Complexes Against Pathogenic Microorganisms

**DOI:** 10.3390/molecules31081367

**Published:** 2026-04-21

**Authors:** Aleksandra Felczak, Katarzyna Niedziałkowska, Katarzyna Lisowska, Urszula Kalinowska-Lis

**Affiliations:** 1 Department of Industrial Microbiology and Biotechnology, Faculty of Biology and Environmental Protection, University of Lodz, 12/16 Banacha Street, 90-237 Lodz, Poland; 2 Department of the Chemistry of Cosmetic Raw Materials, Faculty of Pharmacy, Medical University of Lodz, Muszyńskiego 1, 90-151 Lodz, Poland; urszula.kalinowska-lis@umed.lodz.pl

**Keywords:** danofloxacin metal-based complexes, silver, copper, antibacterial activity, antifungal properties

## Abstract

Fluoroquinolone antibiotics, like danofloxacin, are considered as crucial veterinary drugs due to their high antibacterial potential, a broad spectrum of activity and good pharmacological properties. However, owing to the widespread use of this group of pharmaceuticals, microbial resistance to them is becoming a serious worldwide concern. In the present study, novel silver and copper complexes of danofloxacin were prepared and characterized using ^1^H NMR, ^19^F NMR and IR spectroscopy, ESI-MS spectrometry, and elemental analysis. The antimicrobial properties of the obtained complexes were determined against selected bacterial and fungal strains, including yeast and conidia-forming fungi. Additionally, toxicities of danofloxacin metal-based complex solutions were assessed toward eukaryotic cells. The obtained results indicate that silver(I) and copper(II) complexes of danofloxacin exhibit good antimicrobial activity against bacteria that are important from the veterinary point of view, like *Listeria monocytogenes* or *Campylobacter jejuni*, in concentrations which are not cytotoxic. The MBC values of metal-based danofloxacin complexes for the mentioned strains were 1.5 times lower than those obtained for danofloxacin. Additionally, the solution of the novel silver–danofloxacin complex was found to have a fungicidal effect against the studied *Candida* and *Aspergillus* strains.

## 1. Introduction

Fluoroquinolone antibiotics are a large group of compounds that contain a 4-quinolone system with a fluorine atom in its 6th position. Their mechanism of action is based on the inhibition of DNA gyrase, an enzyme that plays a key role in DNA replication. Fluoroquinolone antibiotics are characterized by a broad spectrum of activity, a long half-life, and excellent bioavailability and these features have contributed to their widespread use in both human and animal therapy. Moreover, in veterinary medicine they are considered as extremely important drugs [1]. An example of these chemotherapeutic agents is danofloxacin (DNX; systematic name: 1-cyclopropyl-6-fluoro-1,4-dihydro-7-[(1S,4S)-5-methyl-2,5-diazabicyclo [2.2.1]hept-2-yl]-4-oxoquinoline-3-carboxylic acid}), which belongs to the third generation of fluoroquinolone antibiotics. Danofloxacin exhibits antibacterial activity against both Gram-positive bacteria (*Staphylococcus aureus*, *Streptococcus* spp.) and Gram-negative strains (*Pasteurella multocida*, *Mannheimia haemolytica*, *Escherichia coli*, *Klebsiella, Salmonella*), mycoplasmas, and *Chlamydia* species [2,3,4]. Apart from its high antimicrobial activity, this compound is also characterized by a long half-life and excellent tissue penetration.

Due to the fact that danofloxacin has been approved for use in all food-producing animals by the EMEA (2002) [5], currently it is commonly applied in the treatment of respiratory and gastrointestinal diseases in cattle, pigs, and poultry [6,7]. It is worth mentioning that danofloxacin is considered as a crucial veterinary antibiotic and is used in the therapy of sick animals and in metaphylaxis [8,9]. The increase in the use of veterinary antibiotics in livestock has contributed not only to environmental pollution, but also to the spread of resistance to these compounds among microorganisms. Due to the decline in the effectiveness of commonly used drugs, it is extremely important to search for new antimicrobial substances to which microorganisms have not yet acquired resistance [9,10].

In recent years, special attention has been paid to the possibility of using complexes of already-known drugs with heavy metals such as silver or copper for this purpose [11]. The antiseptic properties of silver and copper were known already in ancient times, when vessels were made of copper or silver to reduce water spoilage. Compounds containing those metals were often used to treat bacterial infections, an example of which can be silver nitrate or silver sulfadiazine [12,13]. Currently, many metal-based compounds are being synthesized and studied for their antibacterial activity. The literature data indicate that N-heterocyclic compounds, characterized by antibacterial potential, are particularly popular for complexation [14]. It is worth mentioning here that fluoroquinolone antibiotics are based on the quinoline structure, thus constituting an excellent research model for complexation with metals. In the literature, copper complexes with ciprofloxacin or nalidixic acid and structures containing silver and norfloxacin or levofloxacin have been described [13,15].

Literature data indicate that the use of metal antibiotics can be one of the ways to counteract microbial resistance [16,17]. At the same time, research focuses mainly on such antibiotics as ciprofloxacin, levofloxacin, or enrofloxacin. It is worth noting, however, that the literature lacks data on the synthesis, properties, and biological characteristics of complexes of danofloxacin and heavy metals.

In the present work, danofloxacin copper and silver complexes both in the form of solutions and solids were obtained, and their chemical characterization was performed. The biological properties (antimicrobial activity and toxicity) of the solutions were determined. The most common, as well as emerging animal and human pathogens, including *S. aureus*, *E. coli*, *C. jejuni*, *L. monocytogenes*, *P. multicoda*, and *Candida* and *Aspergillus* strains, were used in the research.

## 2. Results

### 2.1. Synthesis and Characterization of Danofloxacin Silver and Copper Complexes

The silver(I) and copper(II) complexes of danofloxacin ligand were prepared by the reaction of danofloxacin with inorganic salts: AgNO_3_ and Cu(NO_3_)_2_∙3H_2_O, respectively, at molar ratios of 2:1.

#### 2.1.1. Danofloxacin–Silver(I) Complex

The obtained solution of the silver(I) complex of danofloxacin was determined using ^19^F NMR spectroscopy and ESI-MS spectrometry. The solid complex was prepared by partial evaporation of the above solution, its filtration, washing with solvent, and drying. The solid complex was characterized by IR and ^1^H NMR spectroscopy and elemental analysis.

The danofloxacin–silver(I) complex with the general formula [Ag(DNX)_2_](NO_3_) was formed. Therefore, its solution contained the silver complex ions [Ag(DNX)_2_]^+^ and the inorganic counterions NO_3_^−^. The ESI^+^-MS spectrometry indicated the presence of two fragmentation peaks: [Ag(DNX)]^+^ and [Ag(DNX)_2_]^+^, which confirms the formation of the Ag(I) complex.

In the ^19^F NMR spectrum only one signal was observed at 135.35 ppm. The ^19^F NMR spectrum of free danofloxacin was also recorded for a comparative purpose and it showed a single peak at 129.05 ppm.

**Figure 1:**
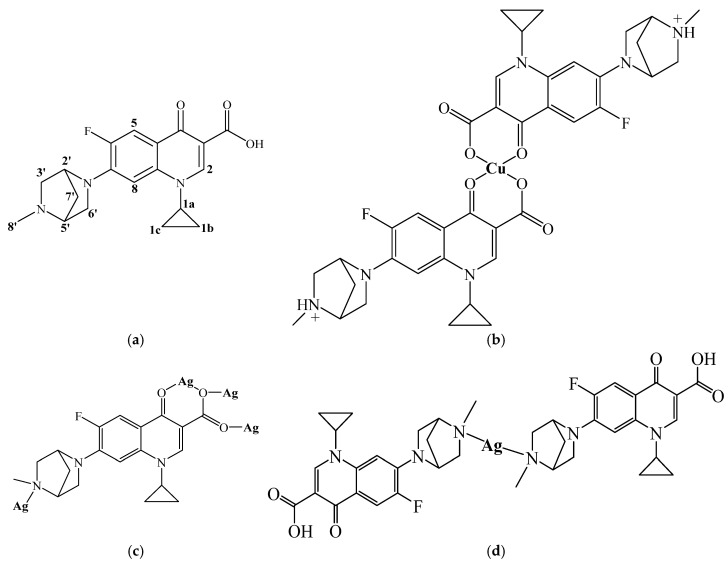
Structural formulas of: (**a**) danofloxacin (DNX) (with atoms’ numbering); (**b**) Cu(II)–DNX complex; (**c**) possible modes of coordination of DNX with silver atom; (**d**) Ag(I)–DNX complex.

Detailed results of ^1^H NMR and IR spectroscopic analysis as well as elemental analysis are provided in Section 4.4.1. The proposed structural formula of the obtained silver(I) complex is shown in Figure 1d.

#### 2.1.2. Danofloxacin–Copper(II) Complex

The obtained solution of the copper(II) complex of danofloxacin was determined on the basis of ESI-MS spectrometry. The solid complex was prepared by partial evaporation of the above solution, its filtration, washing with solvent, and drying. The solid complex was characterized by IR and elemental analysis.

The ESI-MS spectrum indicated the presence of a main molecular ion at *m*/*z* 777 associated with the formation of a danofloxacin–copper(II) complex with the general formula [Cu(DNX)_2_](NO_3_)_2_. Therefore, it can be claimed, the tested solution contained the copper complex ions [Cu(DNX)_2_]^2+^ and the inorganic counterions NO_3_^−^. Under the measurement conditions (ESI-MS), this ion contained copper in the reduced Cu(I) oxidation state, which was probably obtained during electron transfer between the copper(II) complex and a solvent molecule in the gas phase. The reduction of copper from (II) to (I) in the measurement conditions seems to be characteristic for this metal [18,19]. ESI-MS spectrum of the copper complex contained the following peaks at *m*/*z* 598, 777, and 1134, assigned to the following metal–ligand combinations: [Cu(I)_2_(DNX)_3_]^2+^/2, [Cu(I)(DNX)_2_]^+^ and [Cu(I)(DNX)_3_]^+^, respectively. The main peak at 777 fragmented to the following fragmentation ions: 358 [DNX+H]^+^, 420 [Cu(I)(DNX)]^+^, and 598 [Cu(I)_2_(DNX)_3_]^2+^/2.

Detailed results of IR spectroscopic analysis and elemental analysis are provided in Section 4.4.2. The proposed structural formula of the obtained copper(II) complex is shown in Figure 1b.

### 2.2. Antimicrobial Activity of a Selected Metal Complex with Danofloxacin

#### 2.2.1. Antibacterial Properties

Based on the results presented in Table 1, it can be concluded that the tested complexes are active against strains such as *Listeria monocytogenes*, *Campylobacter jejuni*, or *Pasteurella multocida*. Their antibacterial activity is comparable to or even better than the analogous danofloxacin solution. In the case of the *L. monocytogenes* strain, the MBC value determined for both complexes was two times lower than in the system containing danofloxacin. On the other hand, in the *C. jejuni* cultures, the MBC values were determined at a 5000-fold dilution for both the copper and the silver complexes, while the MBC value for the danofloxacin solution was defined in the samples diluted 3000-fold.

#### 2.2.2. Antifungal Activity

Due to the fact that the literature data prove that complexation of drugs with metal can significantly increase the spectrum of activity of the acquired complex, the conducted analyses were also extended to the fungal model.

In the present work, the antifungal activity of the obtained complexes was assessed toward yeasts and conidia-forming fungi. First of all, it should be noted that danofloxacin did not affect the growth of fungi, either yeasts or microscopic filamentous fungi (Table 2 and Table 3).

Both tested danofloxacin complexes were found to demonstrate antifungal activity, wherein the danofloxacin–silver complex exhibited stronger properties. For the aforementioned complex, MIC values were determined in systems diluted 1000-fold and 5000-fold for *C. parapsilosis* and *C. albicans*, respectively. It is worth mentioning that, apart from its ability to inhibit yeast growth, the tested silver and danofloxacin complex was also characterized by fungicidal properties against the tested *Aspergillus* strains (Table 3).

What is very important is that, among the tested compounds, only the danofloxacin and silver complex inhibited spore germination and thus limited the growth of *Aspergillus* strains. MEC values were determined in 500-fold and 100-fold diluted solutions for *A. flavus* and *A. fumigatus*, respectively.

**Figure 2:**
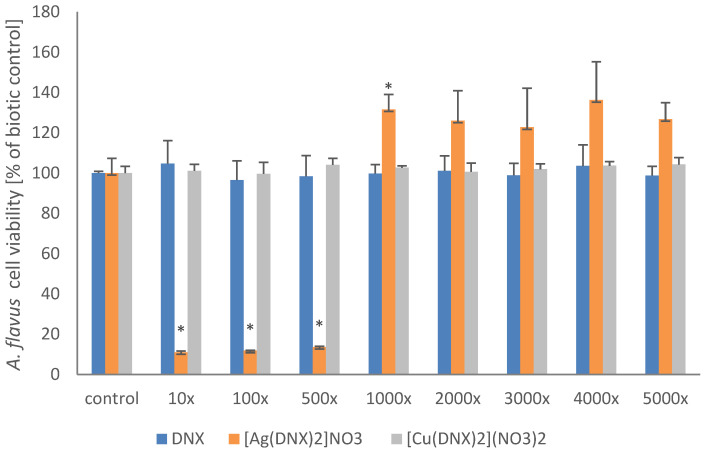
Reduction of the AlamarBlue dye by *A. flavus*. The presented results are expressed as mean ± SD. * denotes statistically significant results (*p* ≤ 0.05) determined by one-way analysis of variance (ANOVA) The dilution ratio of the originally acquired stock solution was determined as follows: 10×, 100×, 500×, 1000×, 2000×, 3000×, 4000×, 5000×^3^.

**Figure 3:**
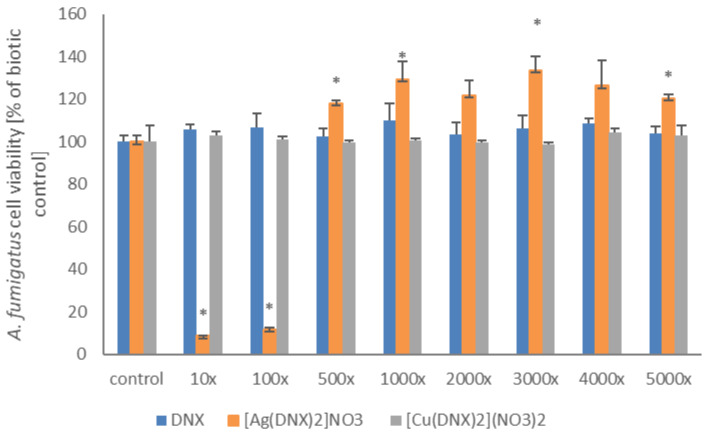
Reduction of the AlamarBlue dye by *A. fumigatus.* The presented results are expressed as mean ± SD. * denotes statistically significant results (*p* ≤ 0.05) determined by one-way analysis of variance (ANOVA). The dilution ratio of the originally acquired stock solution was determined as follows: 10×, 100×, 500×, 1000×, 2000×, 3000×, 4000×, 5000×^3^.

The MEC value determined during tests carried out in accordance with the CLSI standard was additionally confirmed by performing the AlamarBlue test, the principle of which is based on the assumption that only living cells demonstrate the ability to convert resazurin to resorufin. The results are presented in Figure 2 and Figure 3 and are in agreement with previously obtained data. The metabolic activity of the tested filamentous fungi in solutions containing the danofloxacin–silver complex diluted 100-fold and 500-fold for *A. flavus* and *A. fumigatus*, respectively, was negligible in comparison to the control system, which confirms the very good antifungal properties of the discussed system.

### 2.3. Toxicity of Selected Metal Complex with Danofloxacin

#### 2.3.1. Hemolytic Activity of the Solutions of Metal-Based Danofloxacin Complexes

**Figure 4:**
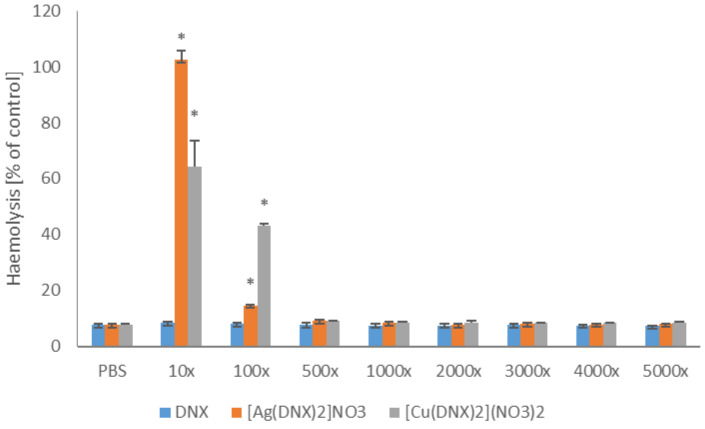
Hemolytic activity of danofloxacin metal based complexes’ solutions in comparison to the parent compound. The presented results are expressed as mean ± SD. * denotes statistically significant results (*p* ≤ 0.05) determined by one-way analysis of variance (ANOVA). The dilution ratio of the originally acquired stock solution was determined as follows: 10×, 100×, 500×, 1000×, 2000×, 3000×, 4000×, 5000×^3^.

In the present work, the toxicity of the tested compounds to human erythrocytes was also determined (Figure 4). The obtained results allow us to state that neither the obtained complexes nor danofloxacin alone caused hemolysis of erythrocytes in dilutions greater than 500-fold. In a solution diluted 100-fold, the hemolytic activity of the silver and danofloxacin complex was comparable to the toxicity shown by the parent substance. However, in the same system, the copper and danofloxacin complex caused hemolysis of 40% of cells.

#### 2.3.2. Cytotoxicity Activity of the Solutions of Metal-Based Danofloxacin

**Figure 5:**
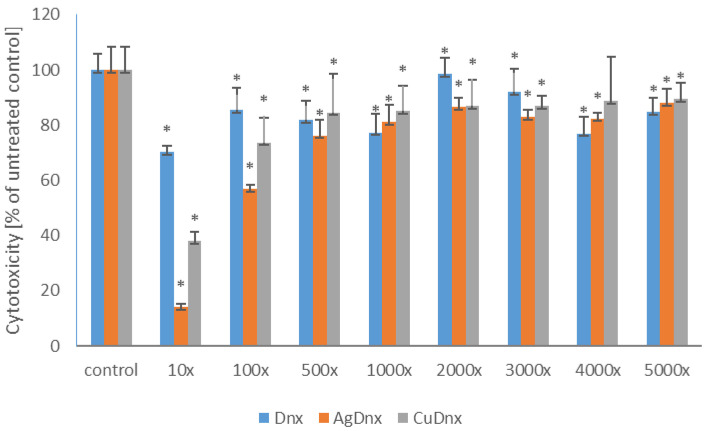
Cytotoxic effect of the solutions of metal complexes with danofloxacin in comparison to the parent compound toward murine fibroblasts. The presented results are expressed as mean ± SD. * denotes statistically significant results (*p* ≤ 0.05) determined by one-way analysis of variance (ANOVA). The dilution ratio of the originally acquired stock solution was determined as follows: 10×, 100×, 1000×, 2000×, 3000×, 4000×, 5000×^3^.

As presented in Figure 5, cytotoxicity of the obtained complexes and danofloxacin was found to be comparable in the range of dilutions from 500-fold to 5000-fold. In the system diluted 100-fold the toxicity of the complexes was higher than that of the parent compound and amounted to 40% and 30% for [Ag(DNX)_2_]NO_3_ and [Cu(DNX)_2_](NO_3_)_2_, respectively.

## 3. Discussion

In the obtained copper(II) complex, the metal ion was coordinated via the pyridone oxygen atom and one of the carboxylate oxygen atoms of danofloxacin, forming a six-membered chelate ring. The terminal piperazinyl nitrogen atom (4′) of the danofloxacin ligand likely exists in the protonated form (CH_3_-NH^+^-) in the complex. A square planar coordination geometry for the copper(II) compound was proposed (Figure 1b). While reviewing the structures of copper(II) complexes with various fluoroquinolones (e.g., ciprofloxacin and norfloxacin), an identical coordination mode of these ligands is observed as in our Cu(II)-DNX complex. Fluoroquinolones act as bidentate ligands coordinated through the oxygen atom of the deprotonated carboxylic group and the carbonyl oxygen atom of the pyridone part of the ligand [20,21,22,23]. In turn, the literature structural data on mixed-ligand copper(II) complexes containing quinolones more often reveal a five-coordinate distorted square pyramidal coordination geometry for the Cu(II) atom, which is related to the type of the second—non-quinolone—ligand [e.g., 2,2′-bipyridine (bipy) or 1,10-phenanthroline (phen)] and the possible coordination of the inorganic counterion [20,21,22].

Studies of silver(I) complexes with fluoroquinolones are less extensive than those of their copper(II) complexes. Fluoroquinolones in Ag(I) complexes can also act as bidentate ligands, or in a totally different way as monodentate ligands, coordinated to the metal ion via the terminal piperazinyl nitrogen (4′). Silver(I)–fluoroquinolone complexes most often adopt T-shaped and linear geometries, less frequently a tetrahedral geometry. The possible modes of coordination of the danofloxacin with silver atom were shown in Figure 1c. It can be coordinated bidentately via the pyridone oxygen atom and one carboxyl oxygen atom, or monodentately via the carboxyl oxygen atoms, or monodentately via the piperazinyl nitrogen (N4′). The structure of the silver complex, in which danofloxacin acts as the bridging ligand, is also probable [21,22,24,25,26,27,28].

In the resulting silver(I) complex, danofloxacin likely acts as a neutral monodentate ligand, which coordinates with silver via the terminal piperazinyl nitrogen atoms (N4′), forming a cationic complex [Ag(DNX)_2_]^+^, neutralized by the nitrate group. The pyridone oxygen atom and one of the carboxylate oxygen atoms of danofloxacin do not participate in coordination with silver (Figure 1d). Other fluoroquinolones, e.g., levofloxacin, ciprofloxacin, and norfloxacin, coordinate with the silver ion in a similar way to our complex [24,25,28].

Fluoroquinolone antibiotics (FQs) have been recognized by WHO as crucial chemotherapeutic agents used in human treatment [29]. Additionally, they are also of great importance in veterinary medicine and are considered as Veterinary Critically Important Antimicrobial Agents [1]. Rising drug resistance, including FQs, is currently considered as the most significant health concern in the modern world [30,31]. This phenomenon affects not only the effectiveness and possibilities of human therapy, but also the health and welfare of livestock, influencing the quality and quantity of food produced [32]. It is known that the acquisition of resistance genes to one of the FQs induces development of antimicrobial resistance to other drugs from this group [8]. Due to the increase in the number of strains resistant to FQs, the EMA and FDA have recommended limiting the use of these drugs. In recent years, there has been noted a decrease in the use of fluoroquinolone antibiotics, but this has not translated into a decrease in the fluoroquinolone resistance rate, which still remains at a high level or is even increasing [33,34]. One of the methods to overcome this phenomenon is to design new active biological molecules based on already-known and described structures. The presented work describes the properties of danofloxacin complexes and selected metals. Silver and copper were used in the studies, because both metals exhibit antibacterial properties and can contribute to the increase in the antibacterial activity of the obtained structures [35,36]. The studied complexes show a comparable or greater activity than danofloxacin in relation to *Listeria monocytogenes*, *Campylobacter jejuni*, or *Pasteurella multocida*. Importantly, *Campylobacter jejuni* and *Listeria monocytogenes* are emerging foodborne pathogens, causing gastroenteritis to serious and chronic infections requiring antibiotic therapy. Mentioned bacteria are detected in different meats and animal products, which is a serious public health concern [37,38]. Also, the *Pasteurella* genus is important from a veterinary point of view, due to its ability to cause numerous infections in humans and animals. *Pasteurella multocida* is an opportunistic pathogen, that is responsible for bovine respiratory disease and pneumonia [39]. Among the above-mentioned bacterial genera, resistance to fluoroquinolones is high and well documented [9,40,41,42]. It is worth emphasizing that the obtained silver and danofloxacin complex is characterized by activity against the tested *Candida* and *Aspergillus* strains, which cause various diseases in both domestic and farm animals from dermatitis and mastitis to pulmonary and gastrointestinal infections [43]. The conducted research also showed that the tested complex has a strong effect on spore germination, a process of key importance in the growth of fungi, conditioning the development of the disease [44]. Additionally, in the concentrations that inhibit the growth of the mentioned microorganisms, the studied danofloxacin metal-based complexes do not exhibit hemolytic or cytotoxic properties.

The results presented in the work are in accordance with the studies of Almehizia et al. [45]. The Authors, who obtained several metal-based complexes with lomefloxacin and pefloxacin, indicated that the Fe(III)–lomefloxacin complex possessed strong antibacterial activity and also inhibited the growth of fungi. The silver complex with levofloxacin was shown to exhibit comparable antibacterial activity as the parent compound and had the ability to limit the growth of *Candida* strains [28]. Also, a metal complex of nalidixic acid with Ag(I) metal was found to possess antifungal properties and limit the growth of microorganisms such as *Pythium aphanidermatum*, *Sclerotinia rolfsii*, *Rhizoctonia solani*, and *Rhizoctonia bataticola* [46]. Seku et al. [47], studied moxifloxacin–Ag(I) metal complexes and indicated that the obtained compounds showed increased antibacterial activity in comparison to the parent drug.

Summing up, complexes of metals and N-heterocyclic ligands are frequently described in the literature, due to their diverse chemical and biological properties. The literature data indicate that factors such as lipophilicity, water solubility, and stability of the complexes are crucial for antimicrobial activity [48,49]. Additionally, complexation of a ligand with documented antibacterial properties and metals, such as silver or copper, may result in an increased antimicrobial activity due to the synergistic effect. The mechanism of action of metal-based complexes is not well understood. Literature data suggest that it may be related to the complexes’ ability to slowly release metal ions, gradually disturb the stability of cell membranes, and interact with microbial proteins. This, in turn, substantially facilitates antibiotic penetration into the microbial cell and reaching their key molecular targets [15,21,48]. The authors also suggest that the ability of some metals to generate ROS, which disrupt the functioning of various metabolic pathways, is also important [15,21,49].

In conclusion, [Ag(DNX)_2_]NO_3_ was characterized by high antimicrobial activity, and apart from the ability to inhibit the growth of bacteria from the genera *Listeria* or *Campylobacter*, was also found to have fungicidal properties against both yeast and conidia-forming fungi.

## 4. Materials and Methods

### 4.1. Reagents and Physical Measurements

Danofloxacin (DNX) and salts: AgNO_3_ and Cu(NO_3_)_2_∙3H_2_O were purchased from Sigma-Aldrich (St. Louis, MO, USA).

^1^H NMR and ^19^F NMR spectra were recorded on a Bruker Avance III 600 MHz spectrometer (Bruker, Billerica, MA, USA) using D_2_O as a solvent. Electrospray mass spectra (ESI-MS) were collected in positive ion mode on a Varian 500-MS LC ion trap (Varian, Palo Alto, CA, USA). The IR spectral data were measured on a Bruker Alpha-T FT-IR spectrometer, and elemental analysis was performed using a Vario Micro Cube by Elemental analyzer (Langenselbold, Germany).

### 4.2. Bacterial and Fungal Strains

Twelve different pathogens were used in the work, including 8 bacteria, 2 species of yeast and 2 strains of microscopic filamentous fungi. The microorganisms used in the study included: *Staphylococcus aureus* ATCC 6358, *Staphylococcus epidermidis* ATCC 12228, *Streptococcus pyogenes* ATCC 19615, *Listeria monocytogenes* ATCC 19115, *Escherichia coli* ATCC 25922, *Pseudomonas aeruginosa* ATCC 15442, *Pasteurella multocida* ATCC 12945, *Campylobacter jejuni* ATCC BAA 1153, *Candida albicans* ATCC 10231, *Candida parapsilosis* ATCC 22019, *Aspergillus flavus* ATCC 9643, and *Aspergillus fumigatus* ATCC 204305.

### 4.3. Cells

Red blood cells needed to determine hemolytic activity were obtained from the Regional Center of Blood Donation and Blood Treatment in Lodz (Poland). NCTC clone 929 murine fibroblasts (ATTC CCL-1) (LGC Standards, Teddington, UK) were used to determine the cytotoxicity of the tested complexes.

### 4.4. Preparation and Charcterization of Metal Complexes with Danofloxacin (DNX)

#### 4.4.1. Preparation of the Silver(I) Complex with Danofloxacin [Ag(DNX)_2_]NO_3_

Danofloxacin (0.5 mmol, 178.7 mg) was added to methanol (ca. 35 mL) at room temperature and a white suspension of the compound was formed. Danofloxacin does not dissolve in methanol even at higher temperature (60 °C). AgNO_3_ (0.25 mmol, 42.5 mg) was dissolved in ca. 30 mL of methanol, and a few drops of distilled water were added to the danofloxacin suspension. The reaction mixture was stirred for 1 h at 40 °C. After a few minutes the cloudy reaction mixture turned into a final clear yellow solution (Figure S1). During the synthesis process the flask with the solution was protected from light with aluminum foil. The final concentration of the prepared solution of [Ag(DNX)_2_]NO_3_ was about 3.40 g/L (approx. 0.43%).

The obtained solution was characterized by ^19^F NMR spectroscopy and ESI-MS spectrometry.

[Ag(DNX)_2_]NO_3_: ^19^F NMR (565 MHz, D_2_O): δ (ppm) 135.35. Danofloxacin (DNX)—for a comparative purpose: ^19^F NMR (565 MHz, D_2_O): δ (ppm) 129.05.

ESI^+^-MS (CH_3_OH) *m*/*z* (relative intensity): 358 (74) [DNX+H]^+^, 464 (25) [Ag(I)DNX]^+^, 715 (97) [2DNX+H]^+^, 821 (100) [Ag(I)(DNX)_2_]^+^, 1177 (13) [Ag(I)(DNX)_3_]^+^ ([Ag(I)(DNX)_2_]^+^ · DNX).

The complex in solid form was isolated by slow evaporation of the above solution at room temperature to approximately 1/4 of the initial volume. The precipitated compound was filtered under vacuum, washed with anhydrous diethyl ether, and dried overnight. The compound was characterized by IR and ^1^H NMR spectroscopy and elemental analysis. MW = 938.66; yield: 185.4 mg (79%).

Anal. Calcd for C_38_H_46_N_7_O_12_F_2_Ag ([Ag(DNX)_2_]NO_3_·3H_2_O): C, 48.62; H, 4.94; N, 10.45%. Found: C, 48.45; H, 4.39; N, 10.50%.

[Ag(DNX)_2_]NO_3_: ^1^H NMR (600 MHz, D_2_O): δ 8.44 (s, 1H, CH(2)), 7.51–7.49 (d, 1H, CH(5)), 6.82 (m, 1H, CH(8)), 4.88–4.77(m, 1H, CH(2′)), 4.32 (s, 1H, CH(5′)), 3.88 (m, 1H, CH(1a)), 3.52–3.31 (m, 4H, CH_2_(6′), CH_2_(3′)), 2.97 (s, 3H, CH_3_(8′)), 2,35 (m, 2H, CH_2_(7′)), 1.26–0.95 (m, 4H, CH_2_(1b), CH_2_(1c)). The atom numbering was shown in Figure 1a.

Danofloxacin (DNX)—for a comparative purpose: ^1^H NMR (600 MHz, D_2_O): δ 8.42 (s, 1H, CH(2)), 7.49–7.47 (d, 1H, CH(5)), 6.76 (s, 1H, CH(8)), 4.83–4.78 (m, 1H, CH(2′)), 4.33 (s, 1H, CH(5′)), 3.88–3.86 (d, 1H, CH(1a)), 3.49–3.31 (m, 4H, CH_2_(6′), CH_2_(3′)), 2.98 (s, 3H, CH_3_(8′)), 2,37–2.30 (m, 2H, CH_2_(7′)), 1.26–0.94 (m, 4H, CH_2_(1b), CH_2_(1c)). The atom numbering was shown in Figure 1a.

Ag(I) complex of DNX: IR (KBr, cm^−1^) ν_max_: 3421(s,br) (OH), 1719(w) (C=O)_COOH_, 1630(s) (C=O)_pyridone_. DNX: IR (KBr, cm^−1^) ν_max_: 3471(s,br) (OH)_COOH_, 1726(s) (C=O)_COOH_, 1630(s) (C=O)_pyridone._

#### 4.4.2. Preparation of the Copper(II) Complex with Danofloxacin [Cu(DNX)_2_](NO_3_)_2_

Danofloxacin (0.5 mmol, 178.7 mg) was added to methanol (35 mL) at room temperature and a white suspension of the compound was formed. Danofloxacin does not dissolve in methanol even at higher temperature (60 °C). Cu(NO_3_)_2_·3H_2_O (60.4 mg), which contained 0.25 mmol, 46.9 mg of anhydrous Cu(NO_3_)_2_ was dissolved in 30 mL of methanol and added to the danofloxacin suspension at room temperature. A clear green solution was formed after combining the substrates. Then the reaction mixture was stirred for 1 h at 40 °C (Figure S1). The final concentration of the prepared solution of [Cu(DNX)_2_](NO_3_)_2_ was 3.47g/L (approx. 0.44%).

The obtained solution was characterized by ESI-MS spectrometry.

ESI^+^-MS (CH_3_OH) *m*/*z* (relative intensity): 358 (12) [DNX+H]^+^, 598 (13) [Cu(I)_2_(DNX)_3_]^2+^/2, 777 (100) [Cu(I)(DNX)_2_]^+^, 1134 (10) [Cu(I)(DNX)_3_]^+^ ([Cu(I)(DNX)_2_]^+^ · DNX). MS^2^ for (777): 358 [DNX+H]^+^, 420 [Cu(I)(DNX)]^+^, 598 [Cu(I)_2_(DNX)_3_]^2+^/2.

The complex in solid form was isolated by slow evaporation of the above solution at room temperature to approximately 1/4 of the initial volume. The precipitated compound was filtered under vacuum, washed with anhydrous diethyl ether, and dried overnight. The compound was characterized by elemental analysis and IR spectroscopy. MW 974.34; yield: 202.2 mg (83%).

Anal. Calcd for C_38_H_48_N_8_O_16_F_2_Cu ([Cu(DNX)_2_](NO_3_)_2_·4H_2_O): C, 46.84; H, 4.96; N, 11.50%. Found: C, 46.60; H, 4.88; N, 11.95%.

Cu(II) complex of DNX: IR (KBr, cm^−1^) ν_max_: 3426 (s,br) (N-H) and (OH)_water_, 1630 (s) (C=O)_pyridone_. DNX: IR (KBr, cm^−1^) ν_max_: 3471(s,br) (OH)_COOH_, 1726(s) (C=O)_COOH_, 1630 (s) (C=O)_pyridone_.

### 4.5. Assessment of Antimicrobial Activity

#### 4.5.1. Antibacterial Activity

The antibacterial activity was determined by the microdilution method in accordance with the Clinical and Laboratory Standards Institute (CLSI) standard M07 (11th Edition) [50] and standard M11 (9th Edition) [51] for antimicrobial susceptibility testing of aerobic and anaerobic bacteria, respectively. The antibacterial properties of the tested strains were determined by the microdilution method, using Mueller–Hinton broth in the case of aerobic bacteria and Brucella medium supplemented with hemin, vitamin K1, and laked horse blood in the model containing anaerobic microorganisms. Incubation of anaerobic bacteria was carried out in jars in which the appropriate atmosphere was provided using Anoxomat Mark II CTS (Mart Microbiology B.V., Drachten, The Netherlands). The samples, biotic and abiotic controls, were incubated for 24 h at 37 °C. The minimal inhibitory concentration (MIC) and minimal bactericidal concentration (MBC) were determined. The MIC value is defined as the lowest concentration of a compound that prevents visible growth of bacteria, while the MBC value defines the lowest concentration of a substance that completely limits the viability of microorganisms.

#### 4.5.2. Antifungal Activity

The antifungal properties of the tested complexes were determined both against yeast and conidia-forming microscopic fungi. Both studies were performed in accordance with the CLSI recommendations Standard M27 (4th Edition) [52] and M38 (3rd Edition) [53] for yeast and conidia-forming fungi, respectively. Both analyses were carried out on the RPMI-1640 medium, and the samples were incubated for 48 h at 37 °C. After this time, the MIC value and minimal fungicidal concentration (MFC), which was defined as the lowest concentration that totally limited the viability of the fungal cells, were determined in the case of yeast. For microscopic conidia-forming fungi, the minimal effective concentration (MEC), the lowest concentration of the tested compounds that disturbs and limits the growth of hyphae in comparison to the hyphal growth visualized in the control well after 2 days of exposure to a drug, was assessed. Additionally, to confirm the lack of growth of the tested fungi and to determine the viability of spores after 48 h of incubation with danofloxacin complexes, the AlamarBlue Assay was performed. The test is based on the ability of living cells to transform resazurin to fluorescent resorufin. The obtained results were presented as a percentage of the biotic control, i.e., a sample containing only microorganism and medium used in the tests.

### 4.6. Toxicity Determination

#### 4.6.1. Hemolytic Activity of the Solutions of Metal-Based Danofloxacin Complexes

Erythrocytes were washed 3 times with PBS and then resuspended in appropriately diluted danofloxacin complex solutions in buffer to achieve a hematocrit of 2.5%. Negative controls (containing PBS) and positive controls (with deionized water) were prepared simultaneously. The samples prepared in this way were incubated in the dark at 37 °C for 24 h. Then the probes were centrifuged at 2800 rpm for 15 min. The degree of hemolysis was determined by spectrophotometric measurement of the hemoglobin released from the erythrocytes into the supernatant. The absorbance of the samples was estimated at λ = 540 nm using a MultiskanTM FC Microplate Photometer (Thermo Fisher Scientific, Pudong, Shanghai, China). The hemolytic activity of the tested complexes was determined using the following formula:

% Haemolysis = ADNXC/APC × 100%

ADNXC is the absorbance of the samples incubated with danofloxacin complexes and APC is the absorbance of the samples containing red blood cells suspended in water.

#### 4.6.2. Cytotoxic Activity of the Solutions of Metal-Based Danofloxacin Complexes

The cytotoxicity test of the solution of metal complexes was carried out using murine fibroblasts, in accordance with the guidelines described in the international standard ISO 10993–5:2009 [54]. Dulbecco’s modified Eagle’s medium (DMEM) containing 10% fetal bovine serum (FBS) with the addition of penicillin/streptomycin (100 IU/100 μg per mL) was used for cell culture. The fibroblast culture was carried out for 24 h at 37 °C, in an atmosphere containing 5% CO_2_. The medium was then removed and the complexes diluted in fresh medium were added to the cell culture. The samples prepared in this way were incubated for another 24 h. After the indicated time, the medium was removed again and the test with 3-(4,5-dimethylthiazol-2-yl) 2,5-diphenyltetrazolium bromide (MTT) was performed. To determine cell viability, a spectrophotometric measurement was performed at λ = 550 nm using a SpectraMax i3x Multi-Mode Microplate Reader (Molecular Devices Ltd., Wokingham, Berkshire, UK). Cell viability was shown as a percentage of the untreated control.

^3^In this work, the solutions of the copper and silver complexes with initial concentrations (Cp) of approx. 0.44% and 0.43%, respectively, were diluted (from 10-fold to 5000-fold) to determine their biological properties (where 100× refers to 100-fold dilution of the stock solution Cp; 500× refers to 500-fold dilution of the stock solution Cp; 1000×—1000-fold dilution of the stock solution Cp; 2000×—2000-fold dilution of the stock solution Cp; 3000×—3000-fold dilution of the stock solution Cp; 5000×—5000-fold dilution of the stock solution Cp). Additionally, all studies on the antimicrobial activity and toxicity of the newly synthesized complexes were referred to the danofloxacin solution containing the same amount of the parent substance as the obtained complexes.

Table 4 shows the concentrations corresponding to the level of dilutions used in the studies.

All biological experiments were carried out in duplicate and the results are presented as the average values of these experiments (n = 4) with the standard deviation. Statistical significance was assessed using a one-way ANOVA test with * *p* < 0.05 and is marked with an asterisk.

## 5. Conclusions

Novel silver(I) and copper(II) complexes of danofloxacin were prepared and characterized using ^1^H NMR, ^19^F NMR and IR spectroscopy, ESI-MS spectrometry, and elemental analysis. The studied solutions of danofloxacin metal-based complexes were found to exhibit good antibacterial activity against bacterial strains which are important from the veterinary point of view (e.g., *Listeria monocytogenes*, *Campylobacter jejuni*, or *Pasteurella multocida*). Additionally, the [Ag(DNX)_2_]NO_3_ complex was characterized as a compound with antifungal properties and the ability to inhibit the growth of fungi of the genera *Candida* and *Aspergillus*. Importantly, the obtained complexes were found to be active in concentrations that are not toxic to eukaryotic cells.

## Figures and Tables

**Table 1:** Antibacterial activity of the solutions of metal-based danofloxacin complexes.

Strain		
	[Ag(DNX)_2_]NO_3_	
	MIC	MBC
*S. aureus* ATCC 6358	˂100×	˂100×
*S. epidermidis* ATCC 12228	˂100×	˂100×
*S. pyogenes* ATCC 19615	˂100×	˂100×
*E. coli* ATCC 25922	˂100×	˂100×
*P. aeruginosa* ATCC 15442	˂100×	˂100×
*C. jejuni* ATCC BAA 1153	5000×	5000×
*L. monocytogenes* ATCC 19115	5000×	1000×
*P. multocida* ATCC 12945	5000×	5000×
	**[Cu(DNX)_2_](NO_3_)_2_**	
	**MIC**	**MBC**
*S. aureus* ATCC 6358	˂100×	˂100×
*S. epidermidis* ATCC 12228	˂100×	˂100×
*S. pyogenes* ATCC 19615	˂100×	˂100×
*E. coli* ATCC 25922	˂100×	˂100×
*P. aeruginosa* ATCC 15442	˂100×	˂100×
*C. jejuni* ATCC BAA 1153	5000×	5000×
*L. monocytogenes* ATCC 19115	2000×	1000×
*P. multocida* ATCC 12945	5000×	5000×
	**DNX**	
	**MIC**	**MBC**
*S. aureus* ATCC 6358	5000×	3000×
*S. epidermidis* ATCC 12228	5000×	5000×
*S. pyogenes* ATCC 19615	3000×	1000×
*E. coli* ATCC 25922	5000×	5000×
*P. aeruginosa* ATCC 15442	3000×	2000×
*C. jejuni* ATCC BAA 1153	5000×	3000×
*L. monocytogenes* ATCC 19115	5000×	500×
*P. multocida* ATCC 12945	5000×	5000×

(The dilution ratio of the originally acquired stock solution was determined as follows: 10×, 100×, 500×, 1000×, 2000×, 3000×, 4000×, 5000×)^3^.

**Table 2:** Antifungal properties of the solutions of metal-based danofloxacin complexes.

Strain	[Ag(DNX)_2_]NO_3_	
	MIC	MFC
*Candida albicans* ATCC 10231	1000×	500×
*Candida parapsilosis* ATCC 22019	5000×	2000×
	**[Cu(DNX)_2_](NO_3_)_2_**	
	**MIC**	**MFC**
*Candida albicans* ATCC 10231	10×	˂10×
*Candida parapsilosis* ATCC 22019	10×	˂10×
	**DNX**	
	**MIC**	**MFC**
*Candida albicans* ATCC 10231	˂10×	˂10×
*Candida parapsilosis* ATCC 22019	˂10×	˂10×

(The dilution ratio of the originally acquired stock solution was determined as follows: 10×, 100×, 500×, 1000×, 2000×, 3000×, 4000×, 5000×)^3^.

**Table 3:** Antimicrobial activity of the solutions of metal-based danofloxacin complexes against conidia-forming fungi.

Strain	[Ag(DNX)_2_]NO_3_
	MEC
*Aspergillus flavus* ATCC 9643	500×
*Aspergillus fumigatus* ATCC 204305	100×
	**[Cu(DNX)_2_](NO_3_)_2_**
	**MEC**
*Aspergillus flavus* ATCC 9643	˂10×
*Aspergillus fumigatus* ATCC 204305	˂10×
	**DNX**
	**MEC**
*Aspergillus flavus* ATCC 9643	˂10×
*Aspergillus fumigatus* ATCC 204305	˂10×

(The dilution ratio of the originally acquired stock solution was determined as follows: 10×, 100×, 500×, 1000×, 2000×, 3000×, 4000×, 5000×)^3^.

**Table 4:** The concentrations of danofloxacin metal-based complexes corresponding to the level of dilutions used in the studies.

Dilution	Concentration [mg/L]		
	[Ag(DNX)_2_]NO_3_	[Cu(DNX)_2_](NO_3_)_2_	DNX
10×	340	350	280
100×	34	35	28
500×	6.8	7	5.6
1000×	3.4	3.5	2.8
2000×	1.70	1.75	1.40
3000×	1.10	1.17	0.93
4000×	0.85	0.88	0.70
5000×	0.68	0.70	0.56

## Data Availability

The data presented in this study are available on request from the corresponding author.

## Supplementary Materials

The following supporting information can be downloaded at: https://www.mdpi.com/article/10.3390/molecules31081367/s1, Figure S1: Photos illustrating the course of the synthesis reaction over time for the danofloxacin-silver(I) complex (a) and the danofloxacin-copper(II) complex (b).
