# Supplementary material for: Antimicrobial Activity of Metal-Based Danofloxacin Complexes Against Pathogenic Microorganisms
Source: Molecules. 2026 Apr 21;31(8):1367. doi: 10.3390/molecules31081367 (PMC13118733; doi:10.3390/molecules31081367)
Supplement: Supplementary file 1 [file molecules-31-01367-s001.zip › molecules-4234464-supplementary.pdf]

SUPPLEMENTARY MATERIALS

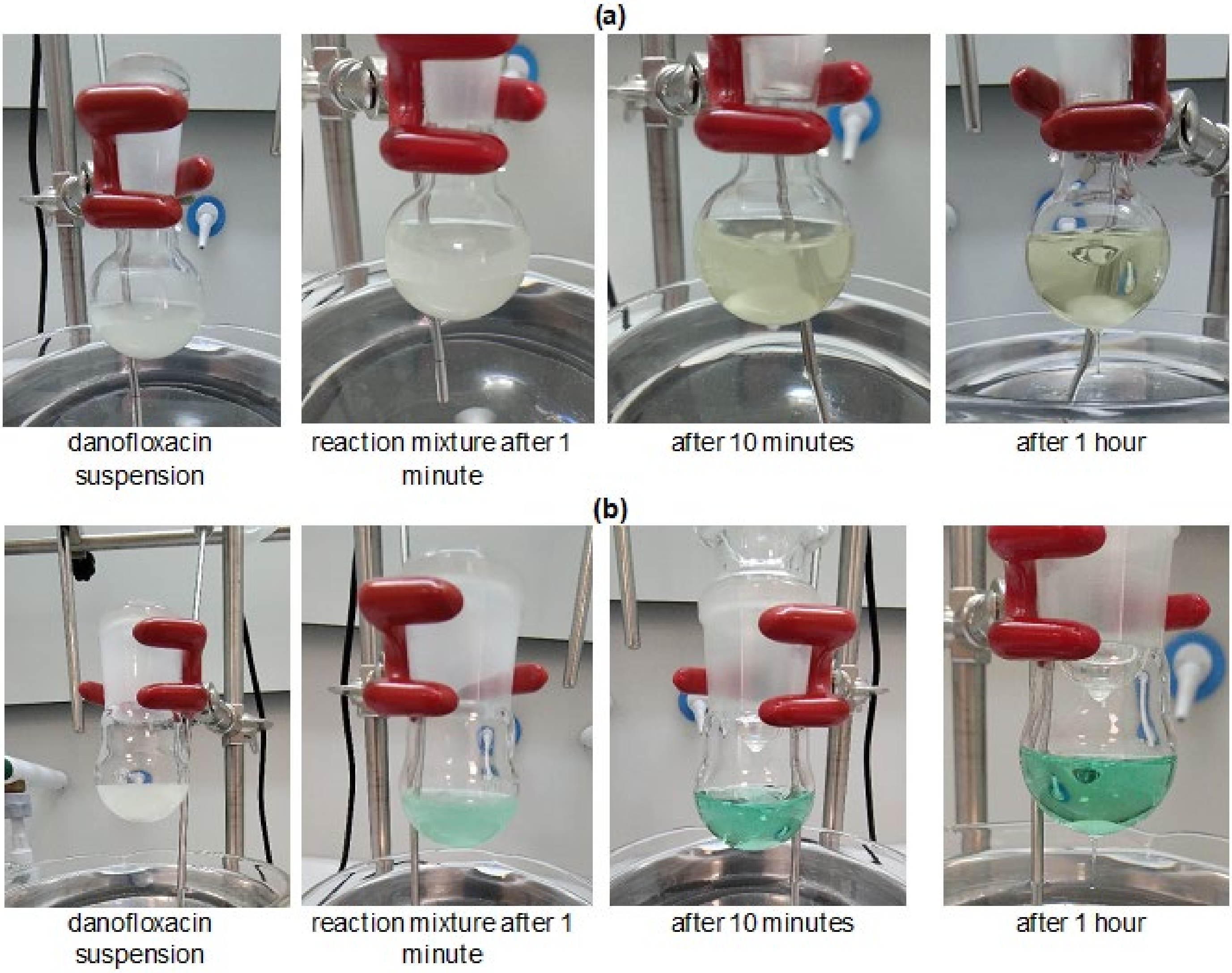

Figure S1. Photos illustrating the course of the synthesis reaction over time for the danofloxacin-silver(I) complex (**a**) and the danofloxacin-copper(II) complex (**b**).
